# MicroRNA-720 suppresses M2 macrophage polarization by targeting GATA3

**DOI:** 10.1042/BSR20160105

**Published:** 2016-08-05

**Authors:** Yan Zhong, Chun Yi

**Affiliations:** * Department of Gynaecology and Obstetrics, The Second Xiangya Hospital of Central South University, Changsha, Hunan 410011, China; † Department of Pathology, Hunan University of Chinese Medicine, Changsha, Hunan 410208, China

**Keywords:** breast cancer, GATA3, macrophage polarization, microRNA-720

## Abstract

Macrophages are highly plastic cells with the ability to differentiate into both M1- and M2-polarized phenotypes. As a distinct M2-polarized population, tumour-associated macrophages (TAMs) promote tumorigenesis owing to their pro-angiogenic and immune-suppressive functions in tumour microenvironment. In the present study, we found that the microRNA-720 (*miR-720*) was down-regulated in TAMs isolated from breast carcinomas and M2-polarization macrophages. Overexpression of *miR-720* attenuated M2 phenotype expression and thus inhibited M2 polarization. We further identified GATA binding protein 3 (GATA3), a transcriptional factor that plays an important role in M2 macrophage polarization, was the downstream target of *miR-720*. Ectopic expression of GATA3 restored the M2 phenotype in *miR-720* overexpressed macrophages. Importantly, overexpression of *miR-720* inhibited pro-migration behaviour and phagocytic ability of M2-polarized macrophages. Thus, our data suggest that *miR-720* plays an important role in regulating M2 macrophage polarization and function.

## INTRODUCTION

Macrophages are highly plastic cells with the ability to differentiate into both M1- and M2-polarized phenotypes [1]. TLR ligands, such as LPS or IFN-γ, induce the polarization of macrophages into M1 phenotype, which promote inflammation and harbour cytotoxic function against tumour cells. However, when macrophages are stimulated with Th2 cytokines, such as IL-4 or IL-13, they are polarized into M2 phenotype, which decrease inflammation and facilitate tissue repair. Tumour-associated macrophages (TAMs) are M2-like macrophages. TAMs actively promote tumour initiation, progression and metastasis due to their immune-suppressive functions in tumour microenvironment [2,3]. Thus, blocking the function of TAMs in tumour microenvironment is a potential therapeutic strategy to inhibit tumorigenesis [4,5].

Several factors regulating macrophage polarization have been identified. For example, transcriptional factors NF-κB, AP-1, and IFN regulatory factor 5 participated in TLR-induced M1 activation, whereas STAT6, C/EBP-β and GATA binding protein 3 (GATA3) were involved in the M2 polarization [6]. In addition, factors involved in epigenetic regulation, including Jumonji domain containing 3 (JMJD3) and Histone deacetylase 3 (HDAC3), also play important roles in M2 macrophage polarization [7,8]. Recently, miRNAs, a new class of non-coding small RNA, have emerged as important regulators in macrophage polarization [9]. Through binding to the UTR of target genes involved in macrophage polarization, miRNAs participate in both M1 and M2 macrophage polarization.

The *miR-720* was firstly identified as a tRNA-derived miRNA [10]. In physiologic conditions, *miR-720* was required for epithelia development and dental pulp cell differentiation [11,12]. Recently, increasing evidence demonstrated that *miR-720* was aberrantly expressed in human cancer and played an important role in tumour progression. For example, in oesophageal squamous cell carcinoma cells, *miR-720* inhibited cell proliferation via targeting p63 [13]. The elevated expression of *miR-720* has been reported in human colorectal cancer and the circulating *miR-720* was identified as a novel serum marker for human malignancies of colorectal cancer [14,15]. However, decreased expression of *miR-720* was reported in breast cancer. Ectopic expression of *miR-720* in breast cancer cells inhibited cell migration by targeting TWIST [16]. Although recent studies suggested important functions of *miR-720* in the development of cancer, the role of *miR-720* in tumour immune microenvironment is largely unknown. In the present study, we investigated the expression and function of *miR-720* in TAMs and found that *miR-720* was required for M2 macrophage polarization by targeting transcriptional factor GATA3.

## MATERIALS AND METHODS

### Reagents

Antibodies to GATA3 (Cat. No. ab106625) and β-actin (Cat. No. ab8226) were purchased from Abcam. Antibodies to human CD86 (Cat. No. 53-0869-41), CD68 (Cat. No. 11-0689-41) and CD163 (Cat. No. 12-1639-41) were purchased from ebioscience. Human IFN-γ, IL-4 and IL-13 were purchased from R&D Systems. PMA and LPS were purchased from Sigma–Aldrich.

### Cell culture

THP-1 and MDA-MB-231 cell lines were purchased from A.T.C.C. THP-1 cells were maintained in RPMI-1640 medium (Invitrogen) and MDA-MB-231 cells were maintained in DMEM medium (Invitrogen) at 37°C with 5% CO_2_ supplemented with 10% fetal bovine serum, 100 units/ml penicillin G, 100 μg/ml streptomycin and 2 mM L-glutamine. Primary monocytes were cultured in RPMI-1640 medium supplemented with 10% (v/v) fetal calf serum (FCS) and 2 mM glutamine, with penicillin (100 units/ml) and streptomycin (100 μg/ml).

### Monocytes differentiation and polarization

For macrophage differentiation, THP-1 cells were treated with 150 nM PMA for 24 h. For macrophage polarization, PMA-treated THP-1 cells were polarized in M1 macrophages by incubation with 20 ng/ml of IFN-γ and 10 pg/ml of LPS for 24 h. Macrophage M2 polarization was obtained by incubation with 20 ng/ml of IL-4 and 20 ng/ml of IL-13 for 24 h.

For human primary monocytes, M1 polarization was induced by treatment of GM-CSF for 6 days and then with LPS (100 ng/ml) and IFN-γ (20 ng/ml) for 24 h. For M2 polarization, monocytes treated with M-CSF for 6 days and then treated with IL-4 (25 ng/ml) for 24 h.

### Patients samples

Breast clinical tumour samples were obtained from the second hospital affiliated to XiangYa, Changsha, Hunan, China. The present study was approved by the Research Ethics Committee of Second Xiangya Hospital, Hunan, China. Informed consent was obtained from all of the patients. TAMs were isolated from 23 fresh tumour samples with a Percoll Density Gradient Centrifugation kit (Pharmacia) as described in [17]. Briefly, the tumour tissues were minced into small pieces and digested with 2 mg/ml collagenase I and 2 mg/ml hyaluronidase at 37° for 2 h. The cells were sequentially filtered through 40 mm cell strainer. The cells were then centrifuged at 2500 rpm for 20 min with 1 ml cell suspension above 5 ml 45% Percoll (GE Healthcare) in the middle and 5 ml 60% Percoll at the bottom in a 15 ml tube. Cells were collected from the cell layer in the interphase between 45% and 60% Percoll. CD14^+^ macrophages were isolated by a magnetic-activated cell sorting using direct CD14 Isolation Kit (Miltenyi Biotec, Cat. No. 130-050-201) according to the manufacturer's instructions. The purity of TAMs was determined by flow cytometry with anti-CD11b and anti-CD14 double staining (Supplementary Figure S1). Monocytes were purified from peripheral blood mononuclear cells (PBMCs) with Monocyte Isolation Kit II (Miltenyi Biotec, Cat. No. 130-091-153) according the manufacturer's instruction.

### Western blotting

Cells were lysed in M2 lysis buffer (150 mM NaCl, 50 mM Tris/Cl (pH 8.0), 5 mM EDTA, 1% Nonidet P40) containing a protease inhibitor mixture (Roche Applied Science) and a phosphatase inhibitor mixture (Sigma). The equal amount of total protein was subjected to SDS/PAGE analysis and immunoblotting with the appropriate antibodies.

### Plasmid construction and transient transfection

The firefly luciferase-expressing vector psiCHECK-2 was used in the luciferase reporter assay. Both wild-type and mutated 3′UTR segment of GATA3 mRNA that contains the putative *miR-720* binding sites was amplified and cloned into the XhoI and NotI sites downstream of the luciferase reporter gene in psiCHECK-2. Lentivirus with *miR-720* expression vector (PLV-720) and control vector (PLV3) were from GenePharma. Briefly, oligonucleotides encoding short hairpin RNA with mature *miR-720* sequences (forward, 5-gatccgtctcgctggggcctccattcaagagatggaggccagcgagacttttttg-3; reverse, 5-aattcaaaaaagtctcgctggcctcctctcttgaatggaggccccagcgaga-cg-3) were subcloned into the restrictive sites BamHI and EcoRI of pGLV3/H1/GFP+ Puro Vector (GenePharma) and verified by DNA sequencing. pFLAG-GATA3 was a gift from Gokhan Hotamisligil (Addgene plasmid # 1419). Following the manufacturer's instructions, plasmids were transfected into cells using Lipofectamine 2000 (Invitrogen). Virus particles were harvested 48 h after cotransfection of PLV-720 or PLV3 with the lentivirus packing vector into HEK-293T cells. *miR-720* overexpressed THP-1 cells were established by infected with PLV-720 lentivirus and selected with puromycin (2 ug/ml) for 48 h.

#### Luciferase assay

For luciferase assay, 0.8 μg of reporter plasmids plus 0.05 μg of pCMV-LacZ vector and PLV-720 or PLV3 empty vector were transfected into the cells using Lipofectamine 2000. At 24 h post-transfection, cells were analysed for luciferase activity. Luciferase assays were performed using the luciferase assay system (Promega); β-galactosidase activity was used as an internal control. Each experiment was conducted a minimum of three times.

### Real-time PCR

TRIzol reagent (Invitrogen) was used to isolate total RNA. The cDNA was prepared by using reverse transcriptase SuperScript III (Invitrogen) with 2 μg of total RNA. Then, 2 μl of the cDNAs was mixed with an 18 μl PCR assay mixture containing 0.5 M each probe and 10 μl Taqman Master Mix (Life Technologies). PCR was conducted with the MyiQ Real-Time PCR Detection System (Bio-Rad Laboratories). The Taqman probe for TNF-α (Assay ID: Hs01113624_g1), IL-6 (Assay ID: Hs00985639_m1), IL-10 (Assay ID: Hs00961620_g1), CCL17 (Assay ID: Hs00171074_m1), Arginase-1 (Assay ID: Hs00968979_m1) and Actin (Assay ID: Hs.PT.56a.21538384) were purchased from Thermo Scientific.

The threshold cycle number for each gene was normalized to that of β-actin, and the resulting value was converted to a linear scale.

Quantitative stem-loop real-time PCR (RT-PCR) was performed to quantify mature *miR-720*. Real-time PCR conducted using SYBR Premix ExTaq (Takara). Sequences of primers for *miR-720* are: forward: 5-gcgtgctctcgctgggg-3; reverse: 5-gcgtgctctcgctgggg-3. snRNA U6 was used as internal controls for miRNA quantification. Sequences of primers for snRNA U6 are: forward: 5-ctcgcttcggcagcaca-3; reverse: 5-aacgcttcacgaatttgcgt-3. All assays were performed at least three times from independent RNA preparations.

### Transwell migration assay

A Transwell migration assay with 6.5-mm-diameter polycarbonate filters (8-μm pore size) was used. Cells (4×10^4^) suspended in 100 μl of DMEM containing 0.5% FBS were seeded in the top chambers. The bottom chambers were filled with 500 μl of DMEM containing 10% FBS. After seeding for 2 h, control or M2-polarized THP-1 cells (5×10^3^) were added into the top chambers. Cells were allowed to migrate for 12 h. Non-migrated cells were removed with cotton swabs, and migrated cells were fixed with cold 4% paraformaldehyde and stained with 1% crystal violet. Images were taken using an inverted microscope (10× magnification; Olympus), and migrated cells were quantified by manual counting.

### Phagocytosis assay

Human THP-1 cells were treated with Staurosporine (1 μm) for 24 h to induce apoptosis and then labelled with CellTracker™ Green (Invitrogen) as target cells. Control or *miR-720* overexpressed THP-1 cells were polarized with IL-4/IL-13 and then labelled with CellTracker™ Red (Invitrogen). For co-incubation, 200 μl of a 2.5×10^5^/ml suspension of target cells was added to each well in 12-well plate, resulting in a 1:1 ratio of macrophage-like cells to target cells. Cells were co-cultured at 37°C in 5% CO_2_ for 30 min. Cells were then washed with PBS for three times and imaged with Zeiss 780 confocal microscope. The phagocytic index was calculated as the ratio of green/red double positive cells to all the red cells in the same field. For each sample, five different fields were measured. The data were presented as three independent experiments.

#### Statistical analysis

Data were analysed using the SPSS (version 20.0; IBM Corporation) software program. Results are expressed as means ± S.D. and are representative of at least three separate experiments. The two-sample *t* test was used to determine statistical differences in the means of two columns. *P* value less than 0.05 was regarded as statistically significant.

## RESULTS

### The expression of *miR-720* is down-regulated in tumour associated macrophages and M2-polarized macrophages

**Figure 1:**
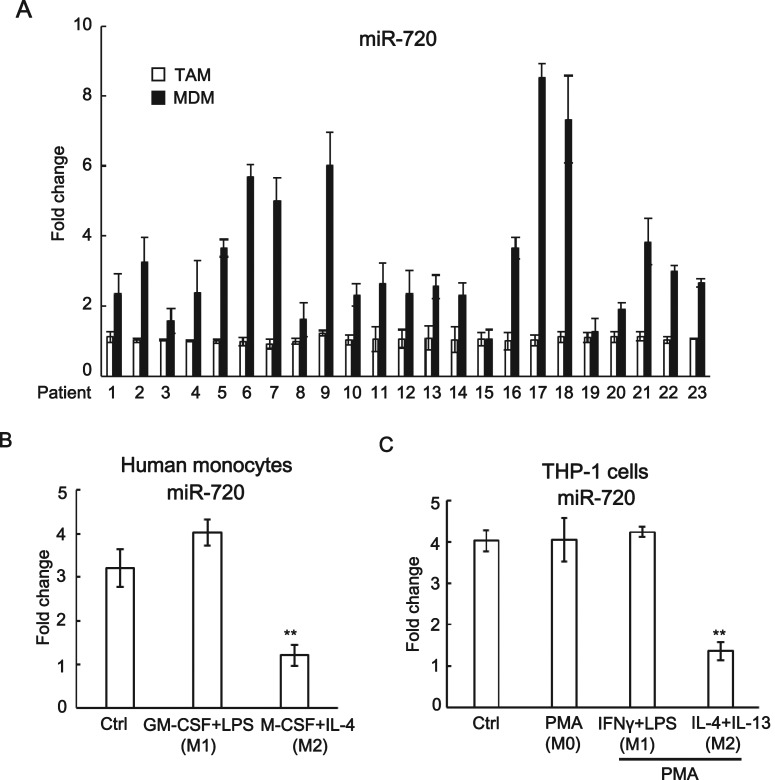
*miR-720* is down-regulated in TAMs and M2-polarized macrophages (**A**) The expression of *miR-720* in TAMs and paired monocyte-derived macrophages from 23 breast cancer patients was analysed by real-time PCR. (**B**) Human monocytes were treated with GM-CSF plus LPS or M-CSF plus IL-4 to induced macrophage differentiation and subsequent M1 or M2 polarization respectively. The expression of *miR-720* was analysed by real-time PCR. (**C**) THP-1 cells were treated with PMA to induce macrophage differentiation. Then, the cells were treated with IFN-γ/LPS or IL-4/IL-13 to induce M1 or M2 polarization respectively. The expression of *miR-720* was analysed by real-time PCR. Values are means ± S.D. ** *P*<0.01 compared with control. The data shown represent three experiments.

To investigate the expression of *miR-720* in TAMs, we isolated TAMs (Supplementary Figure S1) and paired monocyte-derived macrophages (MDMs) from 23 breast cancer patients and examined the expression by quantitative RT-PCR. Interestingly, we found *miR-720* was significantly down-regulated in TAMs compared with the paired MDMs in most of the breast cancer patients (21 out of 23) (Figure 1A). Since TAMs are M2-like macrophages, we then investigated the expression of *miR-720* during M1/M2 polarization of macrophages. The human monocytes were either treated with GM-CSF plus LPS or M-CSF plus IL-4 to induced macrophage differentiation and subsequent M1 or M2 polarization respectively. We found that *miR-720* was also down-regulated in M2 macrophages, although the expression of *miR-720* did not change in M1-polarized macrophages compared with non-differentiated monocytes (Figure 1B). To further confirm our observation, a human monocyte cell line THP-1 cells were differentiated by PMA and subsequently polarized into M1 or M2 phenotype. Similar to our previous observation, the expression of *miR-720* was down-regulated in M2-polarized THP-1 cells, whereas no comparable expression change of *miR-720* was observed among control, PMA-induced (M0) and PMA plus LPS/IFN-**γ-**polarized (M1) THP-1 cells (Figure 1C). Thus, our data indicated that the expression of *miR-720* was specifically down-regulated in TAMs and M2-polarized macrophages.

### *miR-720* suppresses the expression of genes associated with M2 phenotypes

**Figure 2:**
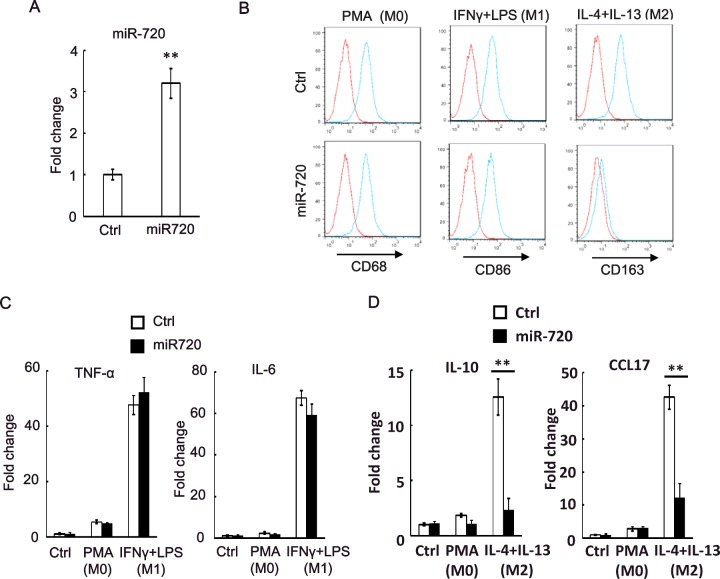
*miR-720* suppresses the expression of M2 phenotypes (**A**) Real-time PCR analysis of *miR-720* expression in control vector or pLV-*miR-720*-infected THP-1 cells. (**B**) Control or pLV-*miR-720*-infected THP-1 cells were induced macrophage differentiation by PMA (M0) and then treated with IFN-γ plus LPS (M1) or IL-4 plus IL-13 (M2) to induce polarization. The macrophage marker CD68, M1 marker CD86 and M2 marker CD163 were analysed by flow cytometry respectively. (**C**) Detection of M1 cytokine TNF-α and IL-6 or (**D**) M2 cytokine IL-10 and chemokine CCL17 by real-time PCR in THP-1 cells used in (**B**). Values are means ± S.D. ** *P*<0.01. The data shown represent three experiments.

To determine whether *miR-720* contributes to the plasticity of macrophage polarization, we overexpressed *miR-720* in THP-1 cells and then induce differentiation and subsequent M1/M2 polarization (Figure 2A). Surprisingly, we found that overexpression of *miR-720* blocked the expression of M2 macrophage marker CD163, whereas overexpression of *miR-720* had no effect on the expression macrophage marker CD68 and M1 marker CD86 (Figure 2B). We then investigated the production of M1- and M2-specific cytokines and chemokines in both control and *miR-720* overexpressed cells. Overexpression of *miR-720* had little effect on the production of M1 macrophage cytokines, TNF-α and IL-6 (Figure 2C), whereas it dramatically inhibited the production of M2 macrophage cytokine, IL-10 and chemokine, CCL17 (Figure 2D). Thus, our data suggested that the expression of *miR-720* is critical for the polarization of macrophage to M2 phenotype.

### *miR-720* targets GATA3 to regulate M2 polarization

**Figure 3:**
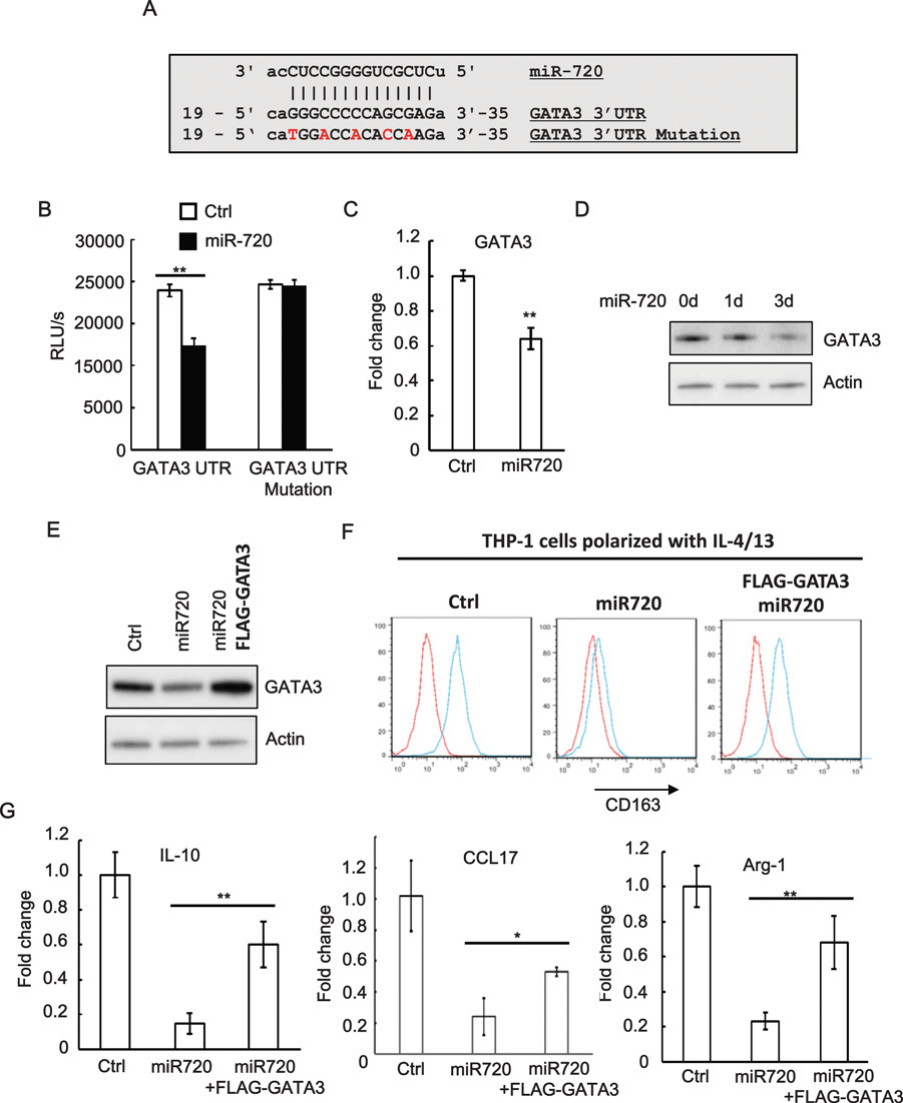
*miR-720* targets GATA3 to regulate M2 polarization (**A**) The 3′UTR of the GATA mRNA containing the putative *miR-720*-binding sequence is shown. Mutation was generated in the GATA 3′UTR sequence at the complementary site for the seed region of *miR-720*. (**B**) HEK-293 cells were co-transfected with wild-type or mutant GATA3 3′UTR luciferase reporter together with control or pLV-*miR-720* construct. Luciferase assays were performed after 24 h. (**C**) The mRNA or (**D**) protein expression levels of GATA3 in both control and pLV-*miR-720*-infected THP-1 cells were analysed by real-time PCR or western blotting respectively. (**E**) Western blotting analysis of GATA3 expression in control, pLV-*miR-720*-infected or pLV-*miR-720*-infected plus FLAG-GATA3 transfected THP-1 cells. (**F**) The cells used in (**E**) were polarized with IL-4/IL-13 and the M2 marker CD163 was analysed by flow cytometry. (**F**) The expression of IL-10, CCL17 and Arginase-1 in the cells used in (**E**) was analysed by real-time PCR. Values are means ± S.D. ** *P*<0.01. The data shown represent three experiments.

We next used two bioinformatics algorithms (TargetScan and microrna.org) to search for the potential targets of *miR-720* involved in M2 polarization. We found GATA3, a transcription factor that has been reported to promote M2 polarization [18], was one of the potential target of *miR-720* on the putative target sequence at 21–32 bp of the GATA3 3′UTR (Figure 3A). We then performed a luciferase reporter assay to determine whether GATA3 is regulated by *miR-720* via direct binding to its 3′UTR. As shown in Figure 3(B), luciferase activity was reduced in the cells co-transfected with luciferase reporter psi-GATA-3′UTR-WT and *miR-720* as compared with the miR control cells. Importantly, this suppressive effect was abolished by mutation of the *miR-720* target sequence (Figure 3B). Consistent with our luciferase data, overexpression of *miR-720* in THP-1 cells reduced expression of GATA3 at both mRNA and protein levels (Figures 3C and 3D). To determine whether GATA3 mediated *miR-720* inhibited M2 polarization, we ectopically expressed GATA3 in *miR-720* overexpressed THP-1 cells (Figure 3E) and we found that ectopic expression of GATA3 restored the expression of M2 macrophage marker CD163 in IL-4 polarized THP-1 cells (Figure 3F). Moreover, the production of M2 cytokine IL-10, chemokine CCL17 and the expression M2 marker Arginase-1 were also partially restored by ectopic expression of GATA3 (Figure 3G). Thus, these data indicated that GATA3 was one of the *miR-720* downstream targets and at least partially mediated suppressive effects of *miR-720* on M2 macrophage polarization.

### *miR-720* regulates cellular functions of M2 macrophage

**Figure 4:**
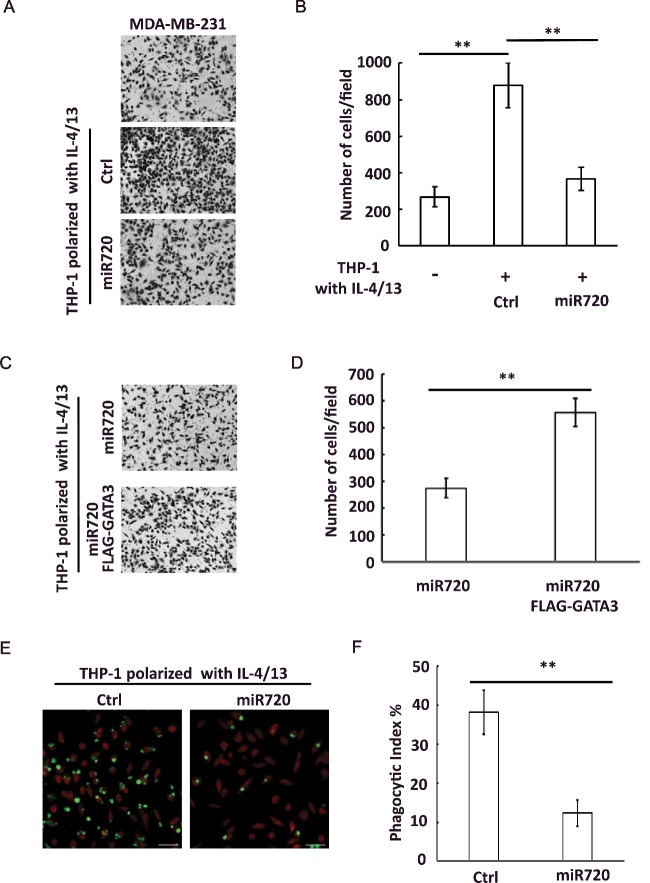
*miR-720* overexpression suppresses potential pro-migration and phagocytic activity in M2 macrophages (**A**) Following the culture of a blank control or the addition of control or *miR-720* overexpressed M2 macrophages derived from THP-1 cells, migrated MDA-MBA-231 cells on the outer surface of the upper chambers were stained with crystal violet (100×). (**B**) Quantitative data of migrated cells from (**A**). Values are means ± S.D. (**C**) MDA-MBA-231 cells were co-cultured with *miR-720* overexpressed or *miR-720* plus FLAG-GATA3 transfected M2 macrophages derived from THP-1 cells. The migrated MDA-MBA-231 cells on the outer surface of the upper chambers were stained with crystal violet (100×). (**D**) Quantitative data of migrated cells from (**C**). (**E**) Apoptotic THP-1 cells were added to control or *miR-720* overexpressed M2 macrophages derived from THP-1 cells and incubated for 30 min. The representative images were shown. (**F**) Phagocytic index was calculated as described in ‘Materials and methods’. Values are means ± S.D. The data shown represent three experiments. **, *P*<0.01.

Since we have demonstrated that *miR-720* suppressed M2 macrophage polarization, we next asked whether *miR-720* regulates cellular functions associated with the M2 phenotypes. It has been demonstrated that M2 macrophages promoted tumour cell migration and invasion [19], which we have confirmed by co-culturing M2-polarized THP-1 cells with MDA-MB-231 breast cancer cells in Transwell system (Figures 4A and 4B). We then evaluated the effect of *miR-720* overexpressed M2 macrophages on the migratory behaviour of breast cancer cells. We found overexpression of *miR-720* in M2-polarized THP-1 cells diminished the ability to promote tumour cell migration (Figures 4A and 4B). To pinpoint whether GATA3 was involved in the inhibitive effect of *miR-720* on tumour cell migration, we ectopically expressed GATA3 in *miR-720* overexpressed THP-1 cells. We found that ectopic expression of GATA3 attenuated inhibitive effect of *miR-720* on tumour cell migration (Figures 4C and 4D). Thus, these data suggested that *miR-720* inhibited pro-migration behaviour of M2-polarized macrophages. Previous studies suggested that M2 macrophages can engulf apoptotic cells more efficient than M1 macrophages [20]. We then evaluated the effect of *miR-720* on the engulfment of apoptotic THP-1 cells by M2-polarized THP-1 cells. We found that overexpression of *miR-720* in THP-1 significantly decreased their ability to uptake apoptotic cells (Figures 4E and 4F). Thus, our data suggested that *miR-720* also inhibited phagocytic ability of M2-polarized macrophages.

## DISCUSSION

Previous study demonstrated that *miR-720* was significantly down-regulated in metastatic breast cancer and inhibited tumour cell migration by targeting TWIST [16]. However, the role of *miR-720* in tumour microenvironment remains elusive. In the present study, we found that the expression of *miR-720* was decreased in TAMs from clinic breast cancer samples. Since TAMs are M2-like macrophages, we further demonstrated that *miR-720* was down-regulated in M2-polarized macrophages. Moreover, functional studies indicated *miR-720* inhibited M2 polarization of macrophages. Currently, there are several miRNAs that have been identified involved in macrophage polarization. For example, let-7c promoted M2 polarization and suppressed M1 polarization by targeting C/EBP-δ [20]. On the other hand, miR-21 impaired M2 polarization while favoured M1 polarization through inhibiting the expression of STAT3 [21]. Remarkably, miR-146a and miR-22 were both down-regulated in TAMs from murine breast cancer [22]. It was demonstrated that miR-146a promoted the expression of M2 macrophage markers, whereas miR-222 inhibited expression of TAM chemotaxis. Our study identified a new specific miRNA to regulate macrophage plasticity. Given the involvement of multiple miRNAs mediating macrophage polarization, it would be interesting to systemically study the functional interaction between *miR-720* and other miRNAs in regulating macrophage plasticity in future.

We then identified GATA3 is the downstream target of *miR-720*, which was supported by multiple lines of evidence: (1) the 3′UTR in GATA3 transcripts contains a *miR-720* binding site; (2) the 3′UTR was responsive to *miR-720* regulation; (3) overexpression of *miR-720* down-regulated GATA3 expression at both mRNA and protein levels. Remarkably, our data suggested that GATA3 mediated inhibitive effects of *miR-720* on M2 polarization since ectopic expression of GATA3 restored expression of M2 marker CD163 in *miR-720* overexpressed THP-1 cells. We noticed that ectopic expression of GATA3 only partially restored other M2 markers such as IL-10 and Arginase-1. These results suggested that other downstream targets of *miR-720* may also contribute to *miR-720* regulated M2 polarization. Interestingly, GATA3 has been identified as a regulator for macrophage polarization. Yang et al. [18] reported that GATA3 was markedly up-regulated in M2 macrophages and loss of GATA3 in mouse monocytes attenuated the expression of several M2 phenotype markers. Therefore, these data suggest that GATA3, at least, is one of the downstream targets of *miR-720* and mediated *miR-720* regulated M2 polarization of macrophage.

TAMs are M2-like macrophages. In tumour microenvironment, TAMs stimulate angiogenesis, promote tumour metastasis and suppress antitumour immune responses. Although previous study reported that *miR-720* inhibited breast cancer metastasis, the function of *miR-720* in TAMs is unknown. Our study indicated that *miR-720* inhibited pro-migration activity of M2 macrophages. Thus, the present study may suggest a novel mechanism how *miR-720* inhibits breast cancer metastasis by targeting TAMs in tumour microenvironment.

Collectively, our study demonstrated that *miR-720* was down-regulated in TAMs and M2-polarized macrophages. We further identified GATA3 was a downstream target of *miR-720* and mediated M2 polarization of macrophages. Importantly, functional study indicated that *miR-720* inhibited potential pro-migration effects of M2 macrophages. Thus, our study reveals a novel mechanism of *miR-720* regulated macrophage polarization and implicates that targeting *miR-720* in TAMs may serve as a promising strategy for breast cancer metastasis.

## Abbreviations

GATA3: GATA binding protein 3
MDM: monocyte-derived macrophage
*miR-720*: microRNA-720
RT-PCR: real-time PCR
TAM: tumour-associated macrophage
